# Photolithographic realization of target nanostructures in 3D space by inverse design of phase modulation

**DOI:** 10.1126/sciadv.abm6310

**Published:** 2022-05-25

**Authors:** Sang-Hyeon Nam, Myungjoon Kim, Nayoung Kim, Donghwi Cho, Myungwoo Choi, Jun Hyung Park, Jonghwa Shin, Seokwoo Jeon

**Affiliations:** 1 Department of Materials Science and Engineering, Korea Advanced Institute of Science and Technology (KAIST), Daejeon 34141, Republic of Korea.; 2 KAIST Institute for NanoCentury (KINC), KAIST, Daejeon 34141, Republic of Korea.

## Abstract

The mass production of precise three-dimensional (3D) nanopatterns has long been the ultimate goal of fabrication technology. While interference lithography and proximity-field nanopatterning (PnP) may provide partial solutions, their setup complexity and limited range of realizable structures, respectively, remain the main problems. Here, we tackle these challenges by applying an inverse design to the PnP process. Our inverse design platform based on the adjoint method can efficiently find optimal phase masks for diverse target lattices and motifs. We fabricate a 2D rectangular array of nanochannels, which has not been reported for conventional PnP with normally incident light, as a proof of concept. With further demonstration of material conversion, our work provides versatile platforms for nanomaterial fabrication.

## Teaser

Advanced phase mask lithography, including photonic inverse designs of mask patterns, realizes target 3D nanostructures.

## INTRODUCTION

The patterning of solid-state materials at the nano- and microscales has offered unprecedented material properties out of their bulk morphologies and performances (*1*–*3*). Owing to their interesting physical and chemical characteristics, three-dimensional (3D) patterned structures have been widely used in many fields, such as photonic/phononic crystals (*4*, *5*), optical/mechanical metamaterials (*6*, *7*), electrochemical platforms (*8*, *9*), and electronic devices (*10*). In parallel, various 3D patterning methods have been studied, including the layer-by-layer method (*11*), controlled hydrogels or aerogels (*12*, *13*), self-assembly methods (*14*), two-photon lithography (*15*), and additive methods (*16*). In particular, serial scanning approaches, such as 3D printing and direct laser writing, have been successfully commercialized, and applications range from medical implants to brake calipers in high-end automobiles. The resolution of the resulting structures in 3D printing can be better than tens of microns; in the case of multiphoton lithography, they can approach 100 nm (*15*, *16*). However, because of their serial nature of operation, they are relatively slow and expensive processes, thus unideal for mass-market products.

**Fig. 1.**
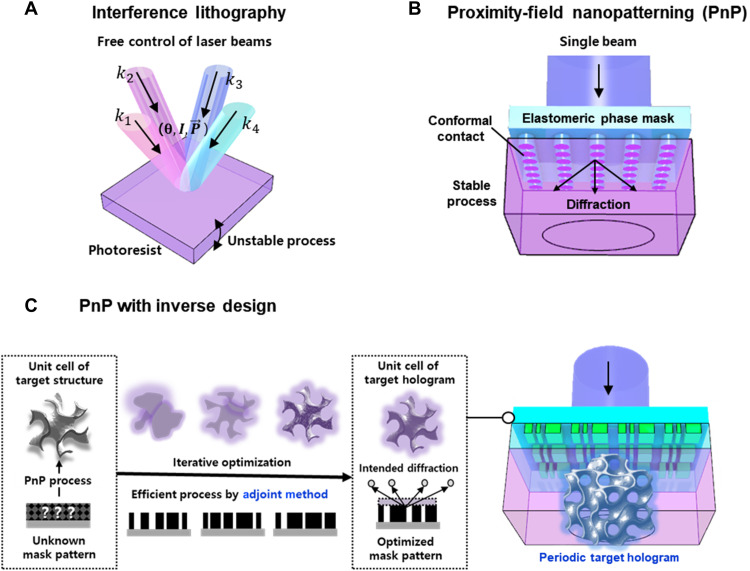
Schematics of 3D holographic lithographies. (**A**) Interference lithography, (**B**) PnP, and (**C**) PnP with inverse design.

Interference lithography can compensate for the drawbacks of additive manufacturing, generating volumetric holographic patterns in a single exposure using a coherent light source (Fig. 1A). This process is fast and precise for producing submicron periodic structures. With proper combinations of four noncoplanar beams with precisely controlled propagation directions, amplitudes, and polarizations, all 14 3D Bravais lattice symmetries can be fabricated (*17*). However, routing multiple beams with such an accuracy requires a complicated optical setup, which is sensitive to environmental conditions, such as thermal drifts or air turbulence.

Proximity-field nanopatterning (PnP) has been suggested as an alternative method for solving issues in interference lithography (Fig. 1B) (*18*–*23*). Instead of preparing multiple incident beams using bulk optics in free space, the PnP process generates multiple beams from a single coherent beam incident on a phase mask that is in conformal and direct contact with the resist to be patterned. Owing to the optical proximity and direct mechanical connection, this method can be less sensitive to environmental fluctuations, resulting in a highly stable and reproducible process. Because only one beam is being controlled, it is relatively easy to extend the process area to a few tens of square inches by the simultaneous extension of areas for the single beam and phase mask pattern (*21*). By adjusting the arrays of grating patterns, different types of 3D lattices such as body-centered tetragonal (BCT) (*20*), hexagonal close-packed (*24*), diamond-like (*25*), and quasicrystal (*19*) lattices can be produced. Different grating parameters, such as the duration and relief depth, allow 3D structures to have different resolutions and feature sizes (*24*). However, simple-grating phase masks have limitations in controlling the diffractions and thus the shapes of the formed structures, which have reduced diversity. To obtain more diverse nanostructures, more complex geometries are required for the phase mask. However, considering the number of design degrees of freedom, the design space is extremely large, and searching for the best design is highly demanding.

In this study, we overcome the limitations of conventional PnP by exploring more complex design spaces in an efficient manner. In particular, we propose an adjoint-based inverse design for the PnP process to fabricate arbitrary target structures. The designed nontrivial grating patterns of the phase mask allow unprecedented optical interference patterns, whereas the fabrication process remains compatible with the established overall process scheme (Fig. 1C). We demonstrate an example of a rectangular lattice with an ellipse motif, which cannot be achieved with conventional PnP, through numerical design optimization and actual fabrication. We also experimentally show that the resulting 3D photoresist nanopatterns with tailored shapes can act as a scaffold for other materials using atomic layer deposition (ALD).

## RESULTS

### Lattice symmetry control by angle-resolved PnP

**Fig. 2.**
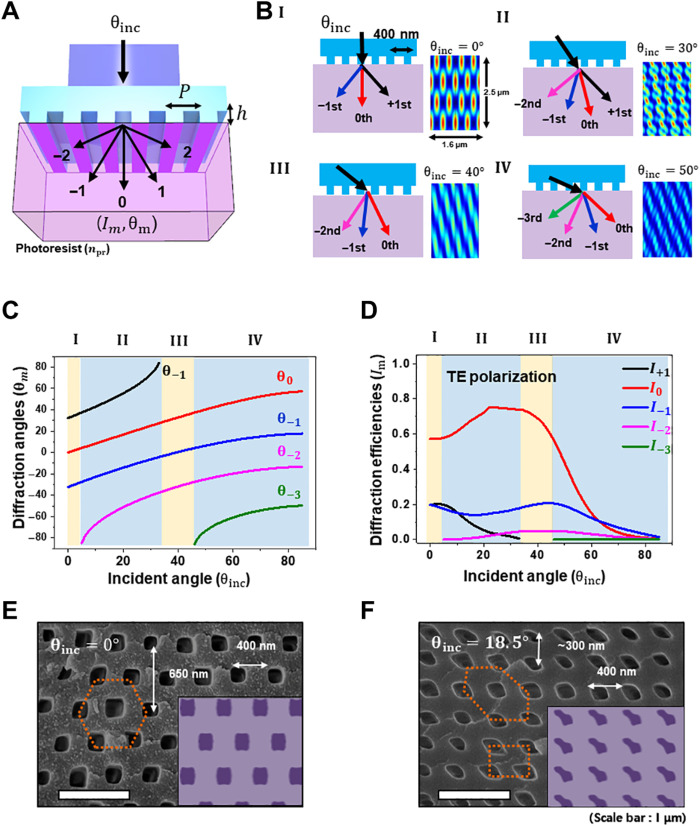
Symmetry control by the PnP process via angle-resolved exposure. (**A**) Schematic of the PnP process with a 1D binary phase mask. (**B**) Diffraction orders and their angles in the photoresist by varying the incident angle with division of four regions according to the number of diffraction orders. (**C**) Diffraction angles of each diffraction order by the exposure angle. (**D**) Diffraction efficiencies of each diffraction order by the exposure angle at TE polarization (*h* = 200 nm). (**E**) Scanning electron microscopy (SEM) cross-sectional image of a hexagonal-array nanochannel via normal exposure (inset: expected structure by FDTD simulation). (**F**) SEM cross-sectional image of a square-array nanochannel via angled exposure (inset: expected structure from FDTD simulation).

The essential factor that defines the structural symmetry in holographic lithography is the incident direction of each interference beam, the relative intensity of which substantially affects the shape of the basis in a unit cell. Similarly, the design of the phase mask pattern and associated lithographic procedures of the PnP technique facilitate the control of diffraction conditions, including the directions and efficiencies of the diffracted beams (Fig. 2A). The number of diffraction orders (*m*) in a photoresist is determined by the equation *m* = [*n*_pr_*P*/λ], and each diffraction angle (θ*_m_*) is calculated as follows

$$\theta_{m}={\text{sin}}^{-1}(\frac{n_{i}\text{sin}\theta_{\text{inc}}}{n_{\text{pr}}}+m\frac{\lambda }{n_{\text{pr}}P})$$ (1)

where λ is the exposure wavelength, θ_inc_ is the incident angle of the exposure light, *P* is the period of the phase mask, and *n_i_* and *n*_pr_ are the refractive indices of the phase mask and photoresist, respectively. The diffraction efficiencies of each beam are defined by not only the wavelength of light but also the geometric factors of the phase mask, such as the period, relief depth, and refractive indices (*24*). Despite technical advances in structural diversity, the PnP technique, which focuses only on normal incident light transmission (θ_inc_ = 0°), has been explored thus far, except for the control of coherence or the minimization of resist absorption for ultrathick (>millimeter) structures (*26*, *27*). This suggests some typical symmetric lattices, such as BCT, diamond-like, and hexagonal lattices, because of the constrained symmetric diffractions (*21*). To expand the classes of symmetries and fundamental optics of PnP, we first adjust the angle of the incident light on a phase mask to generate an asymmetric diffraction pattern (Fig. 2B). The dimension of patterning is set to 2D with a 1D simple-grating phase mask, which shows the basic phenomena of asymmetric diffractions and resultant structures. As the incident angle increases, the diffraction beams are redirected by the horizontal component of the wave vector of the incident light. The diffraction orders occur and disappear according to the relation between the grating vector of the phase mask and the horizontal component of the wave vector. This reveals four stages (Fig. 2B) in the resulting interference, with specific diffraction angles (Fig. 2C) and efficiencies for transverse electric (TE) and transverse magnetic (TM) polarization (Fig. 2D and fig. S2). These numerical results are calculated using rigorous coupled-wave analysis (Gsolver). An additional −2nd diffraction order emerges in region II, where the incident angle θ_inc_ ranges from ~4.72° to ~33.48°. As the three beams interfere and generate lattices in 2D, the redundant 4th beam of the −2nd order breaks periodicity and creates more complex structures. As its efficiency increases with a larger angle of incidence in region II (Fig. 2D and figs. S2 and S3), the simulated pattern implies structural irregularities and complications. A further increase in the incident angle cannot support the +1st order, and only three beams (0th, −1st, and −2nd) remain after θ_inc_~33.48° (region III). The resultant interference patterns and structures become periodic again, as in region I. In the last stage of region IV, the −3rd order is generated and makes complex interference patterns again from θ_inc_~45.73°.

On the basis of the computational design, optics with different incident angles are transferred to the lithography to generate structural examples with 2D symmetries of hexagonal (θ_inc_ = 0°) and square (θ_inc_ = 18.50°) structures (Fig. 2, E and F). The electromagnetic field profiles produced by the PnP process are calculated using a finite-difference time-domain (FDTD) method. Light with a wavelength of 355 nm and a phase mask with a 400-nm periodicity are used to build epoxy-based nanostructures (SU-8 10, Kayaku Advanced Materials) with an exposure dose of approximately 14 mJ/cm^2^ as experimental demonstrations (*21*, *25*). Normally incident exposure induces three diffraction beams of the zeroth and ±1st orders in the symmetric directions (stage I). The resultant hexagonal interference pattern and expected structure are calculated. Notably, this type of symmetry was achieved by following (*28*). As shown in Fig. 2E, the simulation agrees well with the experimental results. When the incident angle is adjusted to 18.50°, asymmetric diffraction is achieved, thereby offering the capability of generating square-array nanochannels via the PnP process (Fig. 2F). Although there are four diffraction beams, a square symmetry is generated with negligible irregularities. This is attributed to the diffraction efficiency of the redundant −2nd order (<1.43%) being lower than that of the other three main orders (1st, 0th, and −1st), making the square symmetric. In this context, the angle-resolved PnP presented here is a straightforward alternative approach to extend the structural diversification with a simple modification of the existing optical setup. Other examples of 2D symmetry demonstrated using angle-resolved methods support technical advances (fig. S3). Although diverse 2D symmetries can be fabricated by angle-resolved PnP, there are still restrictions for modulating the diffraction angles and efficiencies. These variables and the number of orders are determined by diffraction equations to have specific values, indicating that it is impossible to control them independently.

Given these aspects, however, there are limitations regarding the fabrication of arbitrary periodic structures with angle-resolved PnPs. For a phase mask with a simple geometry, the diffraction angles and efficiencies are determined to have specific values. They cannot be controlled independently because the period of the phase mask, refractive index of the polymer, and incident angle affect both simultaneously. These aspects impose constraints on the degrees of freedom of the diffraction phenomena and realizable structures by PnP. It is necessary to find a nontrivial pattern of the phase mask for the generation of the intended diffraction and target hologram.

### Inverse design of phase mask patterns in PnP

We adopt topology optimization to design a phase mask for PnP using the adjoint method. The adjoint method is an efficient way to compute gradients and has been applied to various fields of science and engineering, including mechanics (*29*) and fluid dynamics (*30*). Recently, the adjoint method has gained much attention in photonic device designs, including the design of metalenses (*31*, *32*), metagratings (*33*–*35*), optical switches (*36*), and many other applications (*37*), because gradients can be computed quickly under large numbers of degrees of freedom. Topology optimization with the adjoint method has enabled photonic devices to have complex geometries with enhanced functionalities.

**Fig. 3.**
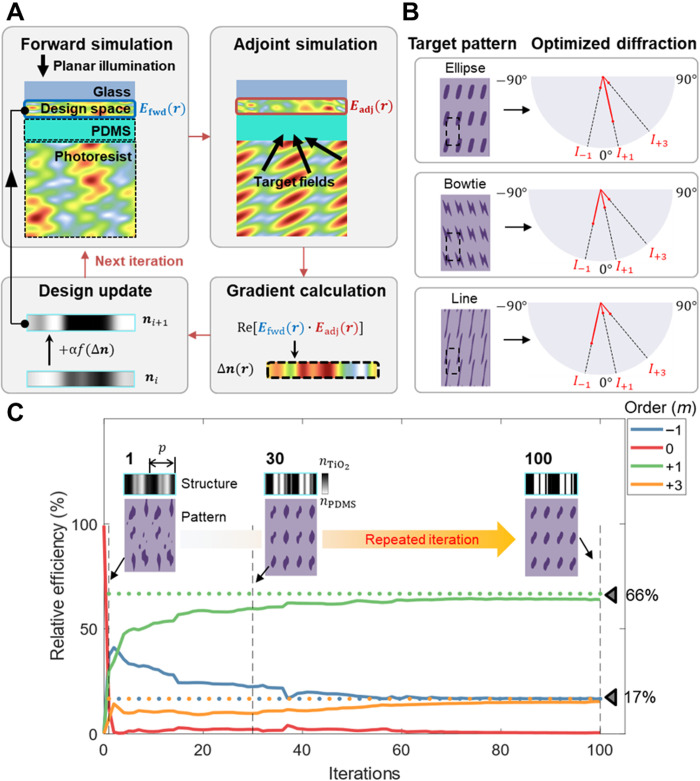
Inverse design of the PnP process. (**A**) One iteration for the gradient-based optimization with the adjoint method. The gradient is calculated on the basis of the interference between the fields in the design space in forward and adjoint simulations. (**B**) Lattice and motif control by the selection and magnitude of each diffraction order, respectively. Rectangular lattices are formed with −1, +1, and +3 diffraction orders. (**C**) Relative efficiencies of the diffraction orders over several iterations. The insets show the gradual evolution of patterns and the corresponding index profile at the 1st, 30th, and 100th iterations.

Our goal is to find a phase mask design that renders the desired interference pattern for a normally incident beam. The physical system of the PnP process is shown schematically in Fig. 3A. The design space is the spatial distribution of materials with different refractive indices that constitute the phase mask, and we assume binary combinations of *n*_PDMS_ = 1.40 and *n*_TiO_2__ = 2.87. A uniform plane wave with a wavelength of 355 nm is incident from the top side. To compute the gradient of a figure of merit (FoM) using conventional numerical simulation methods such as the FDTD method, we need *N* + 1 full-wave simulations, where *N* is the number of design variables. Because *N* is usually large in inverse design problems (from hundreds to millions), performing *N* + 1 simulations requires a very large computational resource. In contrast, by setting the adjoint equation, which uses the symmetry of Maxwell’s equations in a reciprocal system, we can obtain a gradient with only two simulations, regardless of the number of parameters. We record the electric fields inside the phase mask region both in the forward calculation, *E*_fwd_(*r*), and in the adjoint calculation, *E*_adj_(*r*). The forward and adjoint calculations share the same phase mask configuration but are excited by different sources: a normally incident plane wave and the time-reversed version of the desired interference pattern, respectively. After reformulation of the equations, the gradient of the FoM with respect to the index distribution in the phase mask can be calculated by the interference between the forward and adjoint fields in the design space (*38*). Further details of the mathematical expressions for the adjoint method are described in Supplementary Text.

The above steps of the forward and adjoint simulations are iterated during the optimization process. The refractive index at each pixel of the phase mask is treated as a continuous variable with bounds *n*_PDMS_ ≤ *n*(***r***) ≤ *n*_TiO_2__. Starting with a random initial spatial distribution of indices, we gradually improve the phase mask configuration with each iteration. As illustrated in Fig. 3A, one iteration is composed of two simulations (forward and adjoint), a gradient calculation, and a design update. The design update is performed using a typical gradient-based optimization algorithm, such as gradient ascent or Limited-memory Broyden-Fletcher-Goldfarb-Shanno algorithm (L-BFGS). After one iteration, the index distribution of the phase mask is slightly modified to improve the performance with respect to the FoM.

To obtain a desired 2D periodic pattern, the numbers, directions, amplitudes, and phases of multiple beams generated by the phase mask must be precisely controlled. The spatial intensity profile of the multibeam interference can be expressed as (*39*)

$$I(r)=\sum_{m=1}^{N}{|E_{m}|}^{2}+\sum_{m\neq l}^{N}E_{m}^{*}∙E_{l}\text{exp}[i(k_{m}-k_{l})∙r]$$ (2)

where **r** is the position vector, and *E_m_* and **k***_m_* are the complex amplitude and wave vector of the *m*th beam, respectively. To obtain the interference patterns in two dimensions, the difference vectors **k***_m_* − **k***_l_* in Eq. 2 should lie within a common plane for all pairs of *m* and *l*. Mathematically, only three beams are required to form any 2D optical lattice (*39*). All 2D Bravais lattices can be potentially realized with PnP (fig. S4).

As shown in Fig. 2, a simple-grating phase mask would scatter waves in a symmetric fashion with respect to the *z* axis when normally illuminated. Hence, this typically leads to a hexagonal lattice (fig. S5A) unless illuminated by an oblique beam as previously explained (Fig. 2 and fig. S5B). In contrast, asymmetric scattering enables much more diverse interference patterns, such as rectangular lattices, even under normal illumination. Thus, as an illustrative example, we now focus on the formation of a rectangular lattice with an inversely designed phase mask. In the simplest configuration, a rectangular lattice can be formed by three beams propagating at the following angles with respect to the surface normal direction: −1st, 1st, and 3rd (fig. S5C). The period of the phase mask, together with the wavelength of the laser and the refractive index of the photoresist, determines the lattice period of the interference pattern. While further reducing the phase mask period (down to 761 nm in the current setup) results in finer lattice configurations (with lateral lattice period down to 380.5 nm), the decrease in the number of design variables also diminishes the controllability on the shape of the motif. Hence, we set a period of 1 μm for the phase mask for the purpose of demonstration of the working principle.

Even with the same lattice, the motif replicated at each lattice point can be varied by controlling the relative amplitude of each output beam. The morphology of the expected motif is calculated using a simple thresholding model. The threshold intensity can be adjusted to obtain the desired motif shape and size. A properly set threshold intensity, in principle, can induce developed patterns much finer than the diffraction limit provided that the chemistry of the resist and the stability of the laser supporting it (*40*). For example, we find that elliptical, bowtie, and line-segment motifs in a rectangular lattice can be obtained by adjusting the relative intensities of the three beams mentioned above (Fig. 3B, left). These patterns are achieved by setting the target relative scattering efficiencies into the three beams with diffraction orders −1st, 1st, and 3rd as follows: 17, 66, and 17% for the ellipse; 48, 26, and 26% for the bowtie; and 60, 15, and 25% for the line segment, respectively. Moreover, by using four beams including the −3rd order diffraction beam rather than three beams, more diverse patterns, such as a circular shape and a horizontal diabolo pattern, can be created (fig. S14). The relative intensities of the scattered beams shown in Fig. 3B are the actual data obtained from the FDTD simulations of the optimized phase masks. One can confirm that only the beams with the desired propagation angles remain, and scattering toward other angles, including the specular direction, is well suppressed (Fig. 3B, right).

Figure 3C shows the evolution of the phase mask design during optimization for the target example of a rectangular array of ellipses. The FoM for the problem is defined as

$$\text{FoM}=-\sum_{m}w_{m}|T_{m}^{\text{tgt}}-T_{m}^{\text{fwd}}|$$ (3)

where $T_{m}^{\text{fwd}}$ and $T_{m}^{\text{tgt}}$ are the actual and target relative intensities of the *m*th beam, respectively, and their difference is multiplied by a weight factor *w_m_*. Initially, the normally incident beam creates a random interference pattern, as shown in the inset of Fig. 3C. We set $T_{-1}^{\text{tgt}}$ = 0.17, $T_{1}^{\text{tgt}}$ = 0.66, $T_{3}^{\text{tgt}}$ = 0.17, and $T_{n}^{\text{tgt}}$ = 0 (for *n* ∉ { −1,1,3}) to produce an elliptic motif, as shown in Fig. 3B. We use an equal-weight condition (*w_m_* = 1 for all *m*). In each iteration, one forward and one adjoint simulation are conducted as described earlier, after which the design variables are updated by the gradient vector calculated by the recorded forward and adjoint fields. The iteration is repeated 100 times to produce Fig. 3C, which illustrates how the design optimization progresses with repeated iterations for the particular example of an elliptic motif. By choosing an ultraviolet (UV) illumination dose matching the threshold value, a rectangular array of ellipses would form in the resist. We impose the constraints of binary material choice and the minimum achievable linewidth in experiments by applying binary push and pattern blurring filters to the spatial distribution of refractive indices in each iteration (*41*) (see Supplementary Text). With iteration, the slope of the thresholding function applied to the refractive index is increased to make the index distribution effectively binary when the optimization converges to the final structure. The final structure is binarized and has a minimal linewidth of 50 nm, which is the resolution limit of our e-beam patterning equipment. This adjoint-based optimization algorithm produces an optimal structure in much fewer iterations than conventional optimization methods. For example, the best design produced by a conventional genetic algorithm after 200 simulations (the same number of simulations as 100 iterations of the current algorithm) has 20 times more errors (−FoM) in the relative efficiencies between the target and the actual beams (fig. S7).

Because the structure involves high spatial frequencies due to the large deflection angles required, it is difficult to achieve a high-efficiency design in an intuitive way. For example, relying on a generalized Snell’s law and selecting structures pixel by pixel from a structural database made by simulations of a periodic lattice of simple unit cells may work for optical metasurfaces with moderate spatial frequencies, but it would produce unwanted diffraction components and change the relative intensities of the desired beams (*34*). The shape of the motif depends strongly on the relative intensities, as demonstrated earlier. In particular, the undesired scattering in the specular direction can alter the lattice and motif, even if the overall error is less than 3% (fig. S6); thus, an accurate, robust but fast design method, such as the adjoint method, is required.

### Experimental demonstrations of the inverse design of PnP

**Fig. 4.**
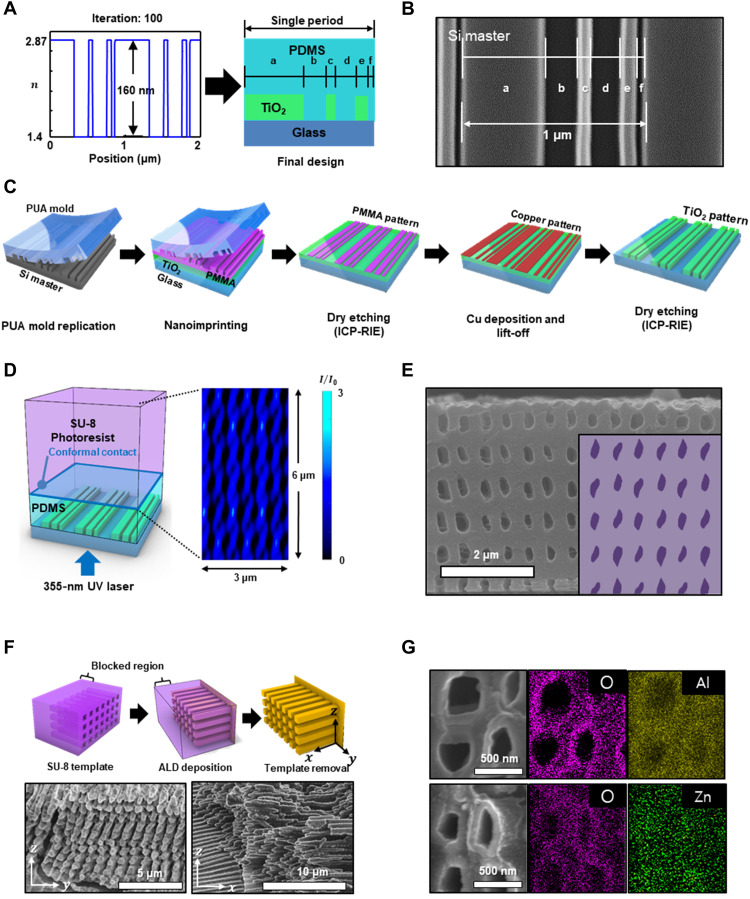
Experimental demonstration of the inversely designed phase mask, fabricated target structure, and material conversion. (**A**) Refractive index profile (left) and a schematic of an inversely designed phase mask consisting of TiO_2_ and PDMS. (**B**) SEM top-view image of Si master with design parameters. (**C**) Fabrication process of the TiO_2_ pattern on a substrate with inverse design parameters. (**D**) A schematic of the exposure process with the inversely designed phase mask (left) and simulation result of the intensity distribution in the SU-8 photoresist (right). (**E**) SEM cross-sectional image of square-array nanochannel obtained via the PnP process (inset: expected structure by FDTD simulation). (**F**) A schematic of the material conversion for the nanochannel (top) and its SEM images with respect to the *yz* and *xz* views (bottom). (**G**) SEM cross-sectional images with energy-dispersive spectroscopy (EDS) for ALD-deposited nanochannels (top, Al_2_O_3_; bottom, ZnO).

The target lattice and motif of the structure are fabricated via the inverse design of the PnP process, and the associated material conversion into functional material reveals its great potential for further applications (Fig. 4). The phase mask relies on the binary structure of the TiO_2_ pattern (thickness of 160 nm) on the poly(dimethylsiloxane) (PDMS) substrate with a period of 1 μm (Fig. 4A). The target 3D architecture for using this inversely designed phase mask is a multilayered 3D nanochannel with a square array through normal incident exposure (355 nm), which has been technically impossible for simple-grating patterns in conventional phase masks. Owing to the phase modulation and conformal contact, the material of the phase mask should be carefully chosen. The building materials for the phase mask with refractive index differences play key roles in achieving both phase shift (∆ϕ = 2π*h*Δ*n*/λ) and the resultant suppression of the zeroth order, which is the transmitted background light (fig. S8) (*21*, *24*). The lower the zeroth-order efficiency is, the higher the image contrast of interference in the photoresist, ensuring an improvement in the structural resolutions (*24*). Considering the exposure wavelength and reliable feature size in lithographic nanofabrication, a phase mask with a small relief depth (*h*) and large refractive index mismatch is required. Therefore, TiO_2_, which has a high refractive index (*n* = 2.87 ± 0.07, *k* = 0.07 ± 0.02 at 355 nm, measured with ellipsometry, alpha-SE, J.A. Woollam), is chosen as the key optical material for efficient phase modulation. The TiO_2_ film is prepared by e-beam evaporation with a deposition rate of 0.5 Å/s and using a rotating substrate. Because the phase mask should be brought into conformal contact with the surface of the prefabricated photoresist film, the TiO_2_ pattern is designed to be embedded in the PDMS supporting matrix (*23*). Although the refractive index mismatch is reduced from 1.87 (TiO_2_-air) to 1.47 (TiO_2_-PDMS), it is still effective in suppressing the zeroth-order efficiency, as shown in fig. S9.

To make this composite phase mask with a stable and reproducible method, we adopt nanoimprinting with a pattern area footprint of ~1 mm by 2 mm with serial nanofabrication (Fig. 4C). The proposed PnP method is as scalable as conventional PnP, which can use stitching or extended-area e-beam lithography to enlarge the processing area (*21*). Briefly, by applying the spatial parameters of the grating (a to f in Fig. 4B) designed by the inverse design (fig. S10), a Si master is fabricated with a depth of ~80 nm by electron beam lithography following dry etching [inductively coupled plasma–reactive ion etching (ICP-RIE)] (Fig. 4B). From this master mold, a UV-curable polyurethane acrylate (PUA) mold is replicated and then applied to align with a polymethylmethacrylate (PMMA; 950 A 2, Kayaku Advanced Materials)/TiO_2_/glass substrate. After annealing the phase mask at 140°C for 15 min to induce PMMA reflow (*T*_g_ of PMMA ≈ 105°C) and cooling at room temperature, the nanoimprinting is completed by detaching the PMMA layer from the PUA mold. Subsequently, the residual layer of the PMMA film is completely etched via ICP-RIE using O_2_ plasma to reveal the TiO_2_ surface. As a hard mask for etching the TiO_2_ film, the Cu pattern is produced by thermal evaporation deposition and lift-off with a thickness of ~10 nm. Owing to the superior selectivity of the ICP-RIE etching technique with CF_4_ plasma, the TiO_2_ pattern maintains its structural stability even after completion of the overall lithographic processes (fig. S10). In addition, the residual Cu is selectively wet-etched using an ammonium persulfate (APS) aqueous solution (2 M) without damaging the TiO_2_ film for less than 1 min. The protecting layer (PDMS) is formed with a thickness of ~100 μm to insulate the TiO_2_ pattern and, more importantly, to allow conformal contact with the photoresist substrate (Fig. 4D). By using the optics of the designed phase mask, the FDTD simulation proves that the intended square-array interference pattern is generated in the SU-8 layer, even with normal incident exposure. The variation in PDMS thickness shows common Fabry-Perot resonance in the diffraction efficiencies with a period of ~140 nm (fig. S11A). When the target diffraction angles and efficiencies of the ±1st and 3rd orders in SU-8 are maintained, as presented in Fig. 3, no notable effect on the interference patterns is observed, except for the shift in the overall images in the direction of the optical axis (fig. S11B). From the comprehensive mask design with the optical simulation, the target square-array nanochannel is obtained in accordance with the computational results (Fig. 4E). Unlike previous results obtained using conventional optics in PnP, the results here are attributed to asymmetric and intended diffractions by subwavelength features in a single period of the phase mask (fig. S12), which enables the fabrication of arbitrary periodic structures with fast, precise, and scalable processes. Actual fabrication processes may involve deviations from ideal configurations, such as dimensional errors in phase mask structures, nonzero linewidth, and temporal drift of the input laser wavelength and intensity. We investigated the sensitivity of the final structures to these deviations to estimate the practicality of the proposed nanofabrication method (fig. S13). The results indicate that the shape and size of the motif, as well as the lattice configuration, are robust against these errors within the level expected in commercially available fabrication equipment.

To incorporate additional material functionality, the nanochannel serves as a scaffold for depositing Al_2_O_3_ layers through ALD (Fig. 4F). As a method to produce vertically arranged nanorods, we prepare a nanochannel array (SU-8) with one end of the sample blocked by intentionally excessive exposure. The detailed preparation for the template includes prior flood exposure, PnP process, and RIE etching (fig. S15). The Al_2_O_3_ layer is then conformally deposited up to 50 nm. The scaffold is thermally etched at 500°C for more than 3 hours. Although the inverse structure has a high nanochannel aspect ratio (channel length, 4 to 20 μm; long-axis diameter, ~450 nm), the robust Al_2_O_3_ nanorods are vertically aligned in square symmetry fixed on the sidewall. Notably, different types of functional materials, including ZnO, can also be prepared, as shown in Fig. 4G, without compromising the structure’s ability to serve as a robust scaffold. To this end, the great potential of this structure includes mainframes of gate-all-around field-effect transistors (*10*), 3D memory (*42*), catalysts (*8*), and sensors (*43*, *44*). Extending the inversely designed dimensions of PnP from 2D to 3D ensures that functional materials defined in arbitrary 3D periodic shapes with nanoscale resolution can be manufactured via a fast, stable, cost-effective, and mass-producible process. This progress is applicable to conventional PnP and provides superior performance in diverse devices (*21*).

## DISCUSSION

Given the large number of design degrees of freedom, the adjoint method enables the phase mask design to be iteratively updated to achieve target diffractions efficiently; hence, the optimized phase mask with a 50-nm feature resolution generates the desired interferences in a photoresist film. The structure realized through the PnP process matched the target pattern and is suitable for mass production. Moreover, we experimentally demonstrated material conversion through ALD and template removal, implying diverse applications in various fields of science and engineering. While the validity of the inverse design approach is demonstrated with interference patterns that are periodic in two dimensions for easier demonstration and explanation, the principle and the computational method are valid for 3D periodic patterns.

## MATERIALS AND METHODS

### ICP-RIE etching process

The dry etching processes for SU-8 templates and TiO_2_ films were conducted by homemade ICP-RIE equipment. The reactive agents for etching SU-8 were SF_6_ [10 standard cubic centimeter per minute (sccm)] and O_2_ (30 sccm) with a total pressure of 10 mtorr. The powers of radio frequency (RF) and bias were 120 and 50 W, respectively. In addition, the total process time was 5 min. The TiO_2_ film was etched to make the intended grating pattern by CF_4_ (30 sccm) gas with a pressure of 10 mtorr. The ICP-RIE powers for RF and bias were 140 and 50 W, respectively. The process time was set to 1 to 3 min. The etching mask was prepared by deposition of Cu by e-beam evaporation on the patterned substrate and lift-off. The Cu mask patterns were removed by APS aqueous solution without damaging SU-8 or TiO_2_.

### Numerical simulation

The desired field patterns were analytically obtained using MATLAB, following Eq. 2. The expected field patterns induced by the phase mask were numerically calculated using the commercial FDTD software (Ansys Lumerical). Field patterns were converted to the pattern by a simple thresholding model, which remain the region below threshold intensity *I*_th_. We used the TM-polarized plane wave for the inverse design. We set the mesh size with λ_0_/150 in phase mask. Far-field diffraction efficiencies in Fig. 3B were obtained using near-to-far-field transformation of the evaluated field at a specific *z* plane in the photoresist. We set periodic condition in the *x* direction and perfectly matched layer in the *z* direction.
