# Supplementary material for: Photolithographic realization of target nanostructures in 3D space by inverse design of phase modulation
Source: Sci Adv. 2022 May 25;8(21):eabm6310. doi: 10.1126/sciadv.abm6310 (PMC9132447; doi:10.1126/sciadv.abm6310)
Supplement: Supplementary file 1 — Supplementary Text Figs. S1 to S15 Table S1 [file sciadv.abm6310_sm.pdf]

Science Advances
AAAS

Supplementary Materials for

Photolithographic realization of target nanostructures in 3D space by inverse design of phase modulation

Sang-Hyeon Nam, Myungjoon Kim, Nayoung Kim, Donghwi Cho, Myungwoo Choi, Jun Hyung Park, Jonghwa Shin*, Seokwoo Jeon*

*Corresponding author. Email: qubit@kaist.ac.kr (J.S.); jeon39@kaist.ac.kr (S.J.)

Published 25 May 2022, *Sci. Adv.* **8**, eabm6310 (2022)
DOI: 10.1126/sciadv.abm6310

The PDF file includes:

Supplementary Text
Figs. S1 to S15
Table S1
Legend for movie S1

Other Supplementary Material for this manuscript includes the following:

Movie S1

Supplementary Text

Relation between crystal lattices and output beam angles

2D periodic lattices can be formed by combining three coplanar beams. Here, we demonstrate the relation between the output beams and the lattices. The three beams passing through the designed phase mask have the same longitudinal wavevector direction as the normally incident beam ($k_z > 0$), as illustrated in Fig. S4. Any two of the differences in $\vec{k_i}$ correspond to the reciprocal lattice vectors (*39*). Without loss of generality, we choose $(\vec{k_2} - \vec{k_1}, \vec{k_3} - \vec{k_2})$ as two reciprocal lattice vectors. The relation between these vectors determines the lattice structures, and the following are the conditions necessary to make all these structures 2D Bravais lattices.

For the rhombic lattice, the two lattice vectors denoted by $\vec{a_1}$ and $\vec{a_2}$ in Fig. S4A should have the same magnitude. For simplicity, if $\theta_2 = 0$, the other beams should have the same magnitudes of k_x and k_z; thus, $|\theta_1| = |\theta_3|$. Therefore, any three symmetric output beams form rhombic lattices. The angle γ has the following relation with θ_3:

$$\gamma = \pi - 2\arctan\left(\frac{\sin\theta_3}{1-\cos\theta_3}\right) \tag{S1}$$

For the hexagonal lattice, which is a special case of the rhombic lattice, the angle between the two lattice vectors $\gamma = \pi/3$. The output angles are thus determined as $\theta_3 = -\theta_1 = \pi/3$ ~~(Fig. S4b)~~.

For the rectangular lattice, the x components of the two lattice vectors should have the same magnitude (Fig. S4B). The relation of the angles between three beams is:

$$\sin\theta_2 - \sin\theta_1 = \sin\theta_3 - \sin\theta_2$$
$$\sin\theta_3 = -\sin\theta_1 + 2\sin\theta_2 \tag{S2}$$

The z components of two of the three beams should be the same ($k_{z1} = k_{z2}$). Therefore,

$$\theta_1 = -\theta_2,$$
$$\sin\theta_3 = -3\sin\theta_1 \tag{S3}$$

For the square lattice, in addition to the rectangular lattice condition, the magnitude of the z component of the lattice vector must be equal to the magnitude of the x component ~~(Fig. S4d)~~. In other words,

$$\sin\theta_3 - \sin\theta_2 = \cos\theta_2 - \cos\theta_3$$
$$\sin\theta_3 + \sin\theta_1 = \cos\theta_1 - \cos\theta_3$$
$$2|\sin\theta_1| = \sqrt{1-\sin^2\theta_1} - \sqrt{1-9\sin^2\theta_1} \tag{S4}$$

By eq. S3, the angle that forms the square lattice is determined as $\theta_1 = \pm\arcsin\left(\frac{1}{\sqrt{10}}\right)$.

The oblique lattice is a general form of 2D periodic lattices. One of the easiest ways to achieve the oblique lattice with three beams is to use two beams symmetric with respect to the z-axis ($\theta_1 = -\theta_2$) and a third beam with the condition $\sin\theta_3 \neq -3\sin\theta_1$(Fig. S4C). For example, if we use the third beam with $\theta_3 = -\arcsin(2\sin\theta_1)$, then the angle γ and ratio between the magnitudes of the two lattice vectors have the following relations (Fig. S4E):

$$\gamma = \arctan\left(\frac{\sin\theta_2}{\cos\theta_2 - \sqrt{2\cos 2\theta_2 - 1}}\right) \tag{S5}$$

$$\frac{|a_1|}{|a_2|} = \frac{2\sin\theta_2}{\sqrt{\left(\cos\theta_2 - \sqrt{-1+2\cos 2\theta_2}\right)^2 + \sin^2\theta_2}} \tag{S6}$$

The interference patterns are formed in real space, and the real lattice vectors $\overrightarrow{b_1}$ and $\overrightarrow{b_2}$ are related to the reciprocal lattice vectors as $\vec{a}_i \cdot \vec{b}_j = 2\pi\delta_{ij}$.

Adjoint method for multiple beam control

The adjoint method allows one to calculate the gradient of the figure of merit (FoM) function with respect to the design variables, only with two simulations. The general introduction of the adjoint method to photonic applications is well described in (*38*), and grating designs for beam control are well explained in the Supporting information of (*34*). Here, we demonstrate that one can selectively obtain beams with certain angles with predefined relative efficiencies.

Our FoM function is the relative efficiencies of beams of different angles. We use the mode overlap integral with a specific angle component. For the mth beam with a specific angle θ_m, the overlap integral is mathematically formulated as

$$\mathrm{T_m^{fwd}} = |\mathrm{t}|^2 = |\mathrm{t}\cdot\mathrm{t}^*| = \left|\iint [\mathbf{E}(\boldsymbol{r}) \times \mathbf{H}_m^-(\boldsymbol{r}) - \mathbf{E}_m^-(\boldsymbol{r}) \times \mathbf{H}(\boldsymbol{r})] \cdot \mathbf{n}\, d\boldsymbol{r}\right|^2 \quad \text{(S7)}$$

where $E_m^-(\boldsymbol{r})$ and $H_m^-(\boldsymbol{r})$ represent the field backward propagation direction with respect to forward calculation.

Now, we perturb a small volume at a specific point **r'** in the design space with $\Delta\epsilon$ to find the effect of material changes.

$$\mathrm{T_m^{fwd'}} = \left|\iint \left[\left(\mathbf{E}(\boldsymbol{r}) + \Delta\mathbf{E}(\boldsymbol{r})\right) \times \mathbf{H}_m^-(\boldsymbol{r}) - \mathbf{E}_m^-(\boldsymbol{r}) \times \left(\mathbf{H}(\boldsymbol{r}) + \Delta\mathbf{H}(\boldsymbol{r})\right)\right] \cdot \mathbf{n}\, d\boldsymbol{r}\right|^2 \quad \text{(S8)}$$

The change in $\mathrm{T^{fwd}}$ can be calculated by subtracting the original one from the perturbed one. Then, the difference is

$$\Delta\mathrm{T_m^{fwd}} = \mathrm{T_m^{\mathit{fwd}'}} - \mathrm{T_m^{fwd}} = 2\mathrm{Re}\left(\mathrm{t}^* \int [\Delta\mathbf{E}(\boldsymbol{r}) \times \mathbf{H}_m^-(\boldsymbol{r}) - \mathbf{E}_m^-(\boldsymbol{r}) \times \Delta\mathbf{H}(\boldsymbol{r})] \cdot \mathbf{n}\, d\boldsymbol{r}\right) \quad \text{(S9)}$$

Using the dipole approximation, the perturbed electric and magnetic fields can be written as:

$$\Delta\mathbf{E}(\mathbf{r}) = \omega^2 \mathbf{G}_{\boldsymbol{e}}(\boldsymbol{r},\boldsymbol{r}')\boldsymbol{p}(\boldsymbol{r}'), \Delta\mathbf{H}(\mathbf{r}) = \omega^2 \mathbf{G}_{\boldsymbol{h}}(\boldsymbol{r},\boldsymbol{r}')\boldsymbol{p}(\boldsymbol{r}')$$

where $\boldsymbol{p} = \epsilon_0 \Delta\epsilon \Delta v \mathbf{E}$ is the dipole moment due to the perturbation. One can reorganize eq. S9 utilizing symmetry and the reciprocity of Maxwell's equation (*38*).

$$\Delta\mathrm{T_m^{fwd}} = 2\omega^2\epsilon_0\Delta\epsilon(\boldsymbol{r}')\mathrm{Re}\left(\mathrm{t}^* \int \left[\left(\mathbf{G}_{\boldsymbol{e}}(\boldsymbol{r},\boldsymbol{r}')\mathbf{E}_m^{\mathrm{fwd}}(\boldsymbol{r}')\right) \times \mathbf{H}_m^-(\boldsymbol{r}) - \mathbf{E}_m^-(\boldsymbol{r}) \times \left(\mathbf{G}_{\boldsymbol{h}}(\boldsymbol{r},\boldsymbol{r}')\mathbf{E}_m^{\mathrm{fwd}}(\boldsymbol{r}')\right)\right] \cdot \mathbf{n}\, d\boldsymbol{r}\right)$$

$$= 2\omega^2\epsilon_0\Delta\epsilon(\mathbf{r}')\mathrm{Re}\left(\mathrm{t}^*\mathbf{E}_m^{fwd} \int [\mathbf{G}_{\boldsymbol{e}}(\boldsymbol{r}',\boldsymbol{r})(-\mathbf{n} \times \mathbf{H}_m^-(\boldsymbol{r}) - \mathbf{G}_{\boldsymbol{h}}(\boldsymbol{r}',\boldsymbol{r})(\mathbf{n} \times \mathbf{E}_m^-(\boldsymbol{r})] \cdot \mathbf{n}\, d\boldsymbol{r}\right)$$

$$= 2\omega^2\epsilon_0\Delta\epsilon(\mathbf{r}')\mathrm{Re}\left(\mathrm{t}^*\mathbf{E}(\boldsymbol{r}) \cdot \mathbf{E}_m^{adj}(\boldsymbol{r})\right) \quad \text{(S10)}$$

where the adjoint field is defined as

$$\mathbf{E}_\mathrm{m}^\mathrm{adj} = \int [\mathbf{G}_{\boldsymbol{e}}(\boldsymbol{r}',\boldsymbol{r})(-\mathbf{n} \times \mathbf{H}_m^-(\boldsymbol{r}) - \mathbf{G}_{\boldsymbol{h}}(\boldsymbol{r}',\boldsymbol{r})(\mathbf{n} \times \mathbf{E}_m^-(\boldsymbol{r})] \cdot \mathbf{n}\, d\boldsymbol{r} \quad \text{(S11)}$$

Therefore, we obtain the gradient $\frac{\partial \mathrm{T_m^{fwd}}}{\partial \epsilon}$ by calculating interference of the field distribution for forward simulation and adjoint simulation in the design space.

We can easily extend single beam control to multiple beam control. Now, our final FoM is defined as in eq. (1) in the main text. The gradient of this summation can be represented as:

$$\frac{\partial \mathrm{FoM}}{\partial \epsilon} = \sum_m w_m 2\epsilon_0 \omega^2 \Delta V Re\left(t_m^* \mathbf{E}_m^{\mathrm{fwd}}(\boldsymbol{r}') \cdot \mathbf{E}_m^{\mathrm{adj}}(\boldsymbol{r}')\right) \quad \text{(S12)}$$

Since $\mathbf{E}_m^{\mathrm{fwd}}(\boldsymbol{r}')$ is identical regardless of beam m, we can reformulate the equation as:

$$\frac{\partial \mathrm{FoM}}{\partial \epsilon} = 2\epsilon_0 \omega^2 \Delta V Re\left(\mathbf{E}^{\mathrm{fwd}}(\boldsymbol{r}') \cdot \left(\sum_m w_m t_m^* \mathbf{E}_m^{\mathrm{adj}}(\boldsymbol{r}')\right)\right) \quad \text{(S13)}$$

Using the linearity of Maxwell's equations, the gradient of multiple beams can be calculated with two simulations as well.

<u>Auxiliary filters for geometric constraints</u>

We use the density design variable $0 \leq \rho \leq 1$. Topology optimization iteratively updates the density of each "bit" of the variable to maximize the FoM values. To meet the conditions of experimental realizations, we need to enforce the constraints using filters.

Minimal linewidth constraints exist due to the fabrication resolution. Since one bit in our design space has a length of 5 nm, we apply a window averaging filter (*41*).

$$\widetilde{\rho_i} = \frac{\sum_{j \in N_i} w_{ij} \rho_j}{\sum_{j \in N_i} \rho_j} \quad \text{(S14)}$$

Here, w_{ij} is the weight of averaging, and we choose a triangle function for the weights:

$$\mathrm{w_{ij}} = \max\left(R - \left|r_i - r_j\right|, 0\right) \quad \text{(S15)}$$

We average with the near region using a triangular window function. We choose R=50 nm as the minimal linewidth corresponding to the resolution of e-beam lithography.

On the other hand, a binary push filter requires the structures to have a refractive index of either $\mathrm{n_{PDMS}}$ or $\mathrm{n_{TiO2}}$. We use the tanh function for filtering, which is described as:

$$\bar{\rho}_i = \frac{\tanh(\beta\eta) + \tanh\left(\beta(\widetilde{\rho_i} - \eta)\right)}{\tanh(\beta\eta) + \tanh\left(\beta(1 - \eta)\right)} \quad \text{(S16)}$$

The hyperparameters β and η control the binarization intensity and the threshold value, respectively. We initially set η as 0.5 and β=1.0 and occasionally update β by 20% during the optimization processes.

Finally, the actual refractive indices during the optimization can be calculated by applying two filters serially and performing interpolation afterward. The refractive index at each pixel can be represented as

$$\mathrm{n_i} = \bar{\rho}_i \cdot n_{\mathrm{PDMS}} + (1 - \bar{\rho}_i) \cdot n_{\mathrm{TiO_2}} \quad \text{(S17)}$$

Our formalism of the adjoint method is based on the relative permittivity. Thus, we use the relation between the refractive index and permittivity, $\mathrm{n_i} = \sqrt{\epsilon_i}$.

Both the binary push filter and the minimal linewidth filter are differentiable functions; thus, we can calculate the gradient using the chain rule:

$$\frac{\partial \rho}{\partial n} = \frac{\partial \rho}{\partial \tilde{\rho}} \cdot \frac{\partial \tilde{\rho}}{\partial \bar{\rho}} \cdot \frac{\partial \bar{\rho}}{\partial \epsilon} \cdot \frac{\partial \epsilon}{\partial n} \quad (S18)$$

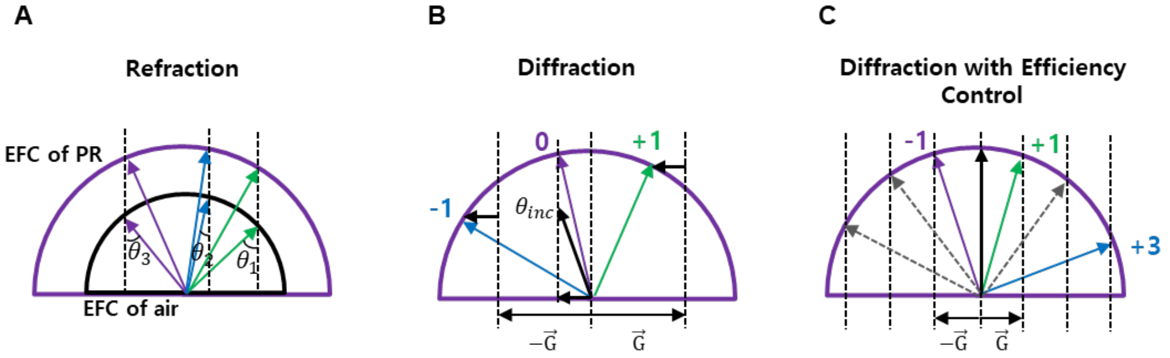

Fig. S1. Equi-frequency contours of each patterning process. (**A**) refraction at the boundary of photoresist and air in interference lithography. (**B**) Diffraction in the photoresist film by phase mask grating. (**C**) Diffraction with control of each order's efficiency via inverse design of phase mask pattern.

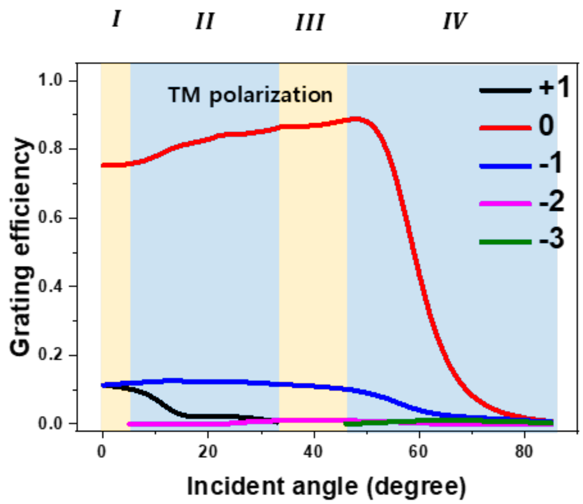

Fig. S2. Diffraction efficiencies of the PDMS phase mask (period: 400 nm, relief depth: 200 nm) at a 355 nm wavelength. Variation of the incident angle in the transverse magnetic (TM) polarization mode.

.

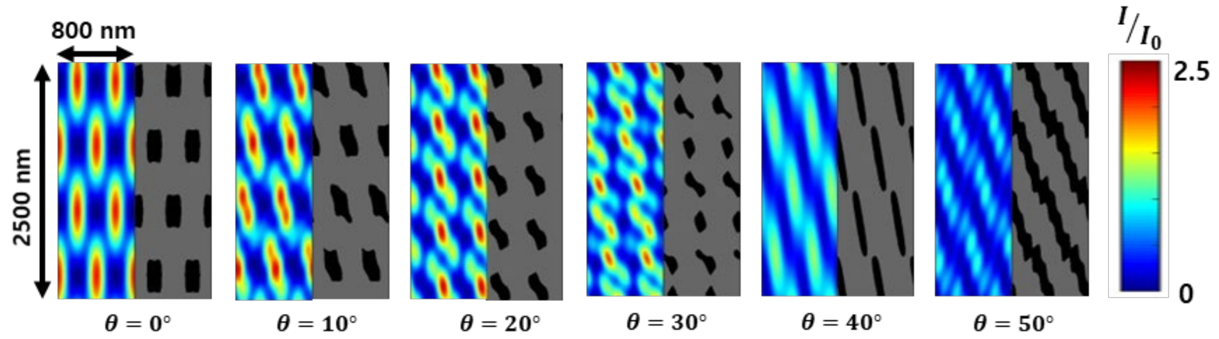

Fig. S3. Intensity distributions in photoresist films and corresponding expected structures after PnP process with repect to incident exposure angles.

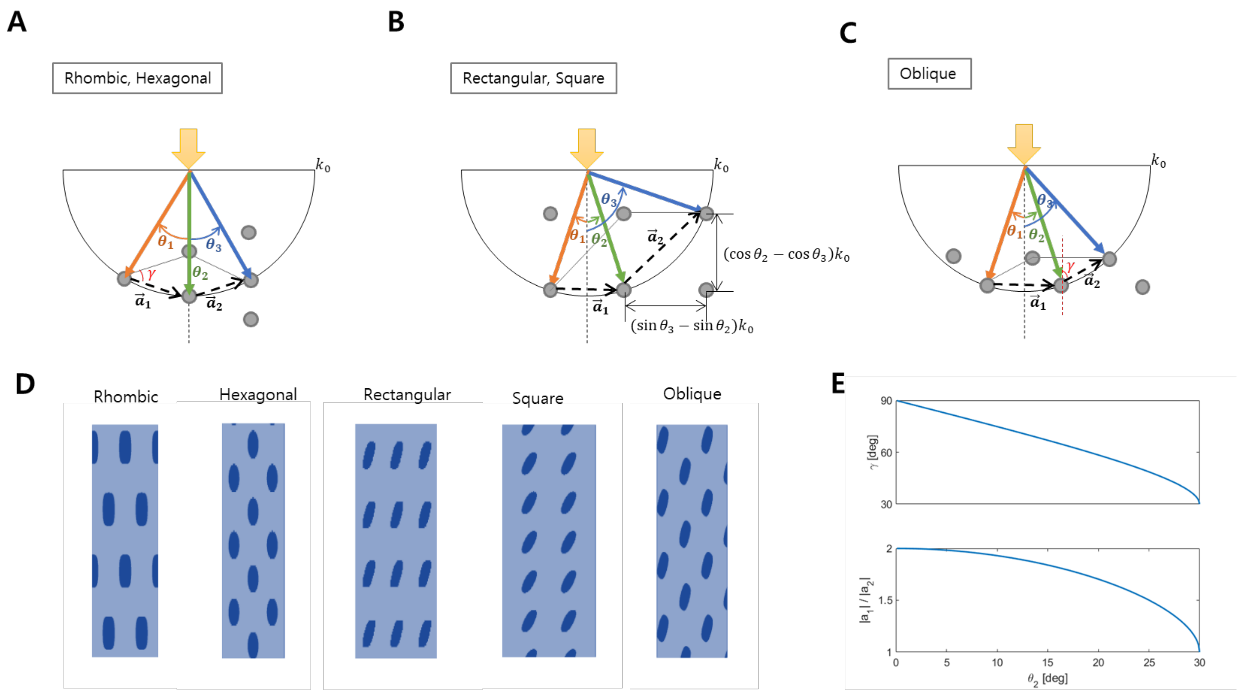

Fig. S4. Realization of all 2D Bravais lattices. (**A-C**) Lattice formation with 3 beams with different angles. (**D**) Analytic calculation of periodic structures. (**E**) Angle between lattice vectors and amplitude ratio for oblique structures by varying θ_2.

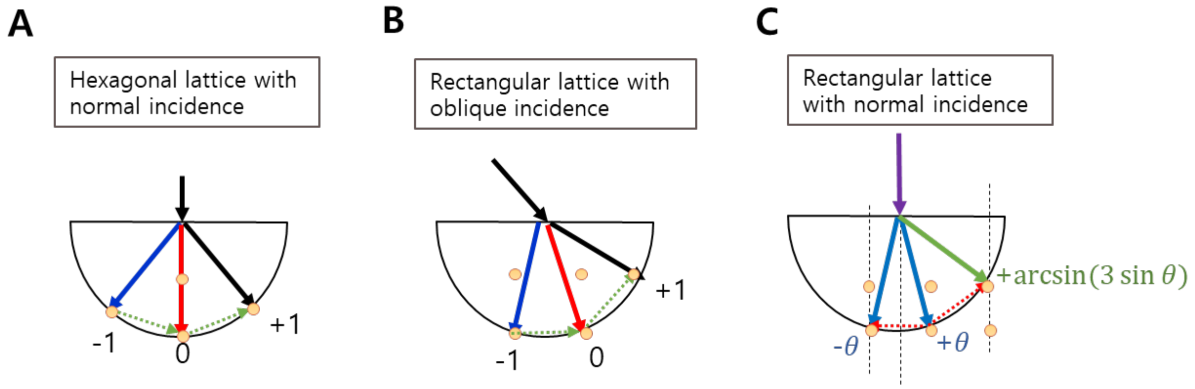

Fig. S5. Hexagonal and rectangular lattice formation by angle-resolved and inversely designed PnP.

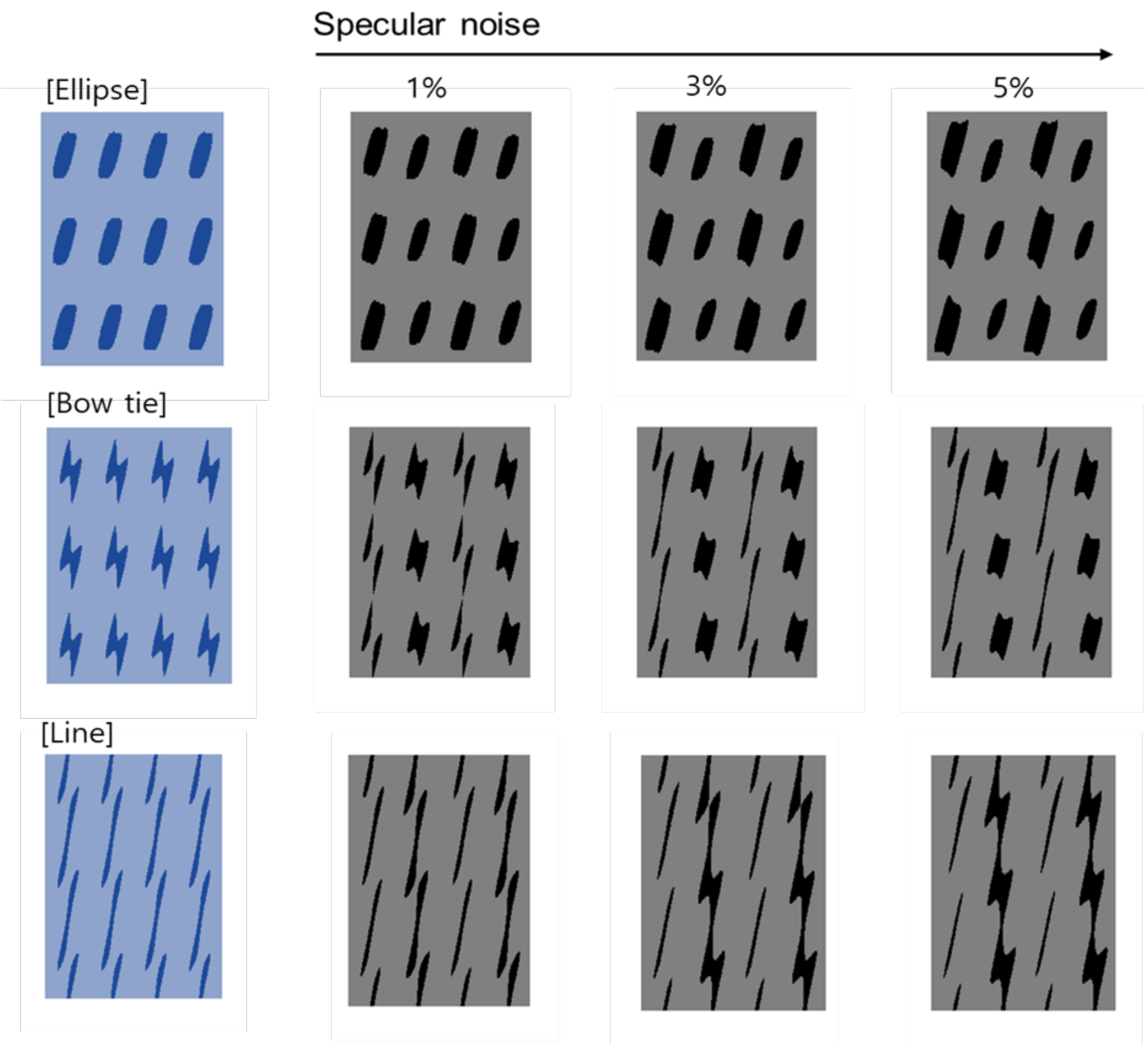

Fig. S6. The effect of undesired beam components

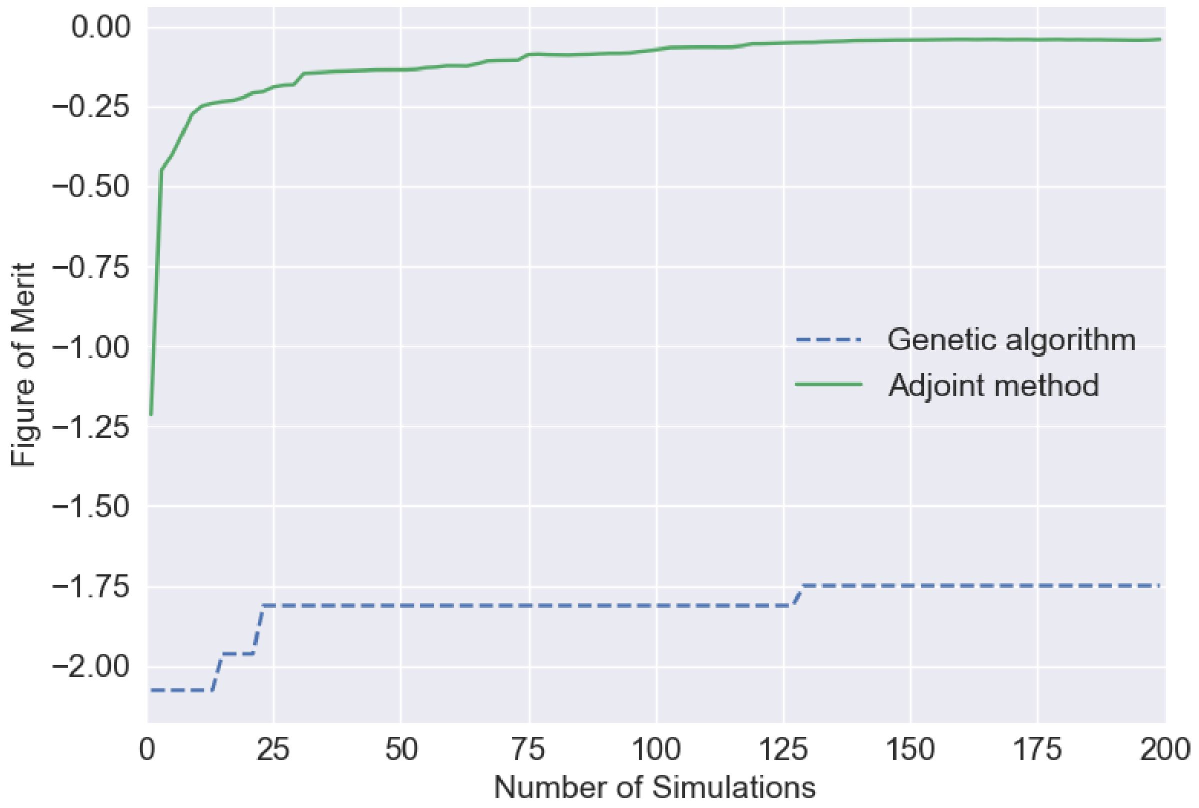

Fig. S7. Comparison of the optimization speed and performance between the adjoint-method-based optimization and genetic algorithm.

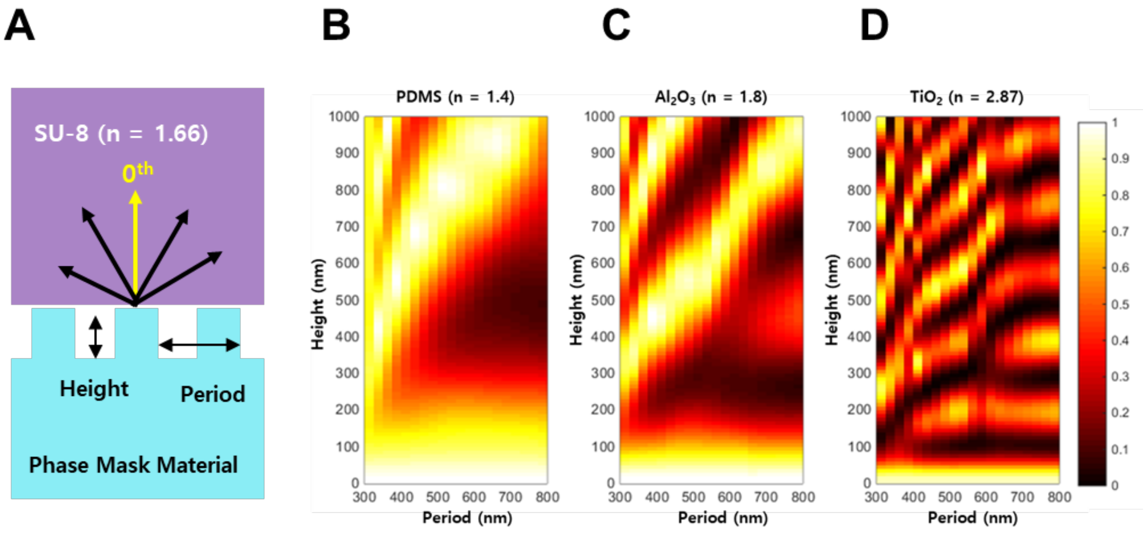

Fig. S8. Diffraction efficiencies of 0th order in SU-8 photoresist as variations of material, period, and height of phase mask at the wavelength of 355 nm. (**A**) Schematics of PnP process and diffraction. 0th order efficiencies as the materials of phase mask; (**B**) PDMS, (**C**) Al_2O_3, and (**D**) TiO_2.

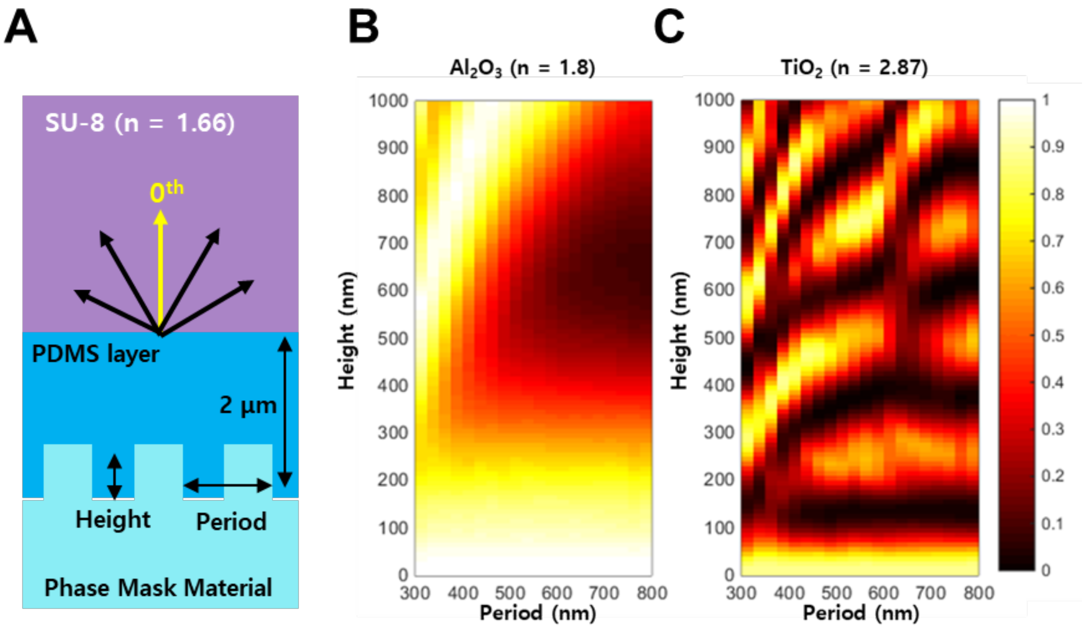

Fig. S9. Diffraction efficiencies of 0th order in SU-8 photoresist as variations of material, period, and height of composite phase mask at the wavelength of 355 nm. (**A**) Schematics of PnP process and diffraction. 0th order efficiencies as the materials of phase mask; (**B**) PDMS-Al_2O_3, and (**C**) PDMS-TiO_2.

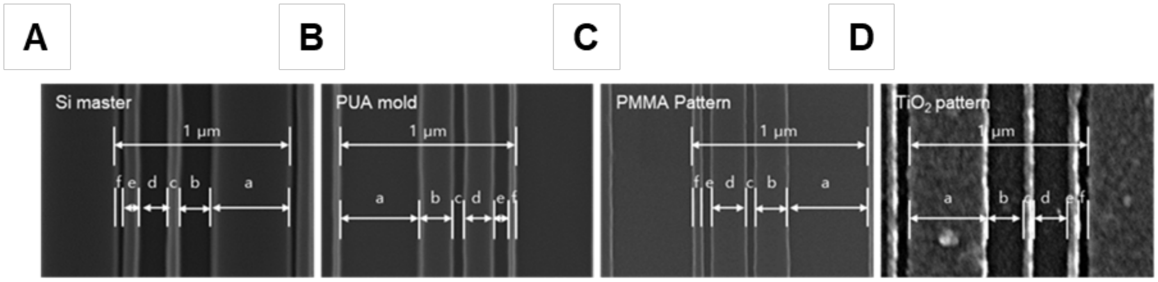

Fig. S10. SEM top-view images. (**A**) the silicon master, (**B**) replication of the PUA pattern from the Si master, (**C**) the imprinted PMMA pattern, and (**D**) the dry-etched TiO_2 pattern.

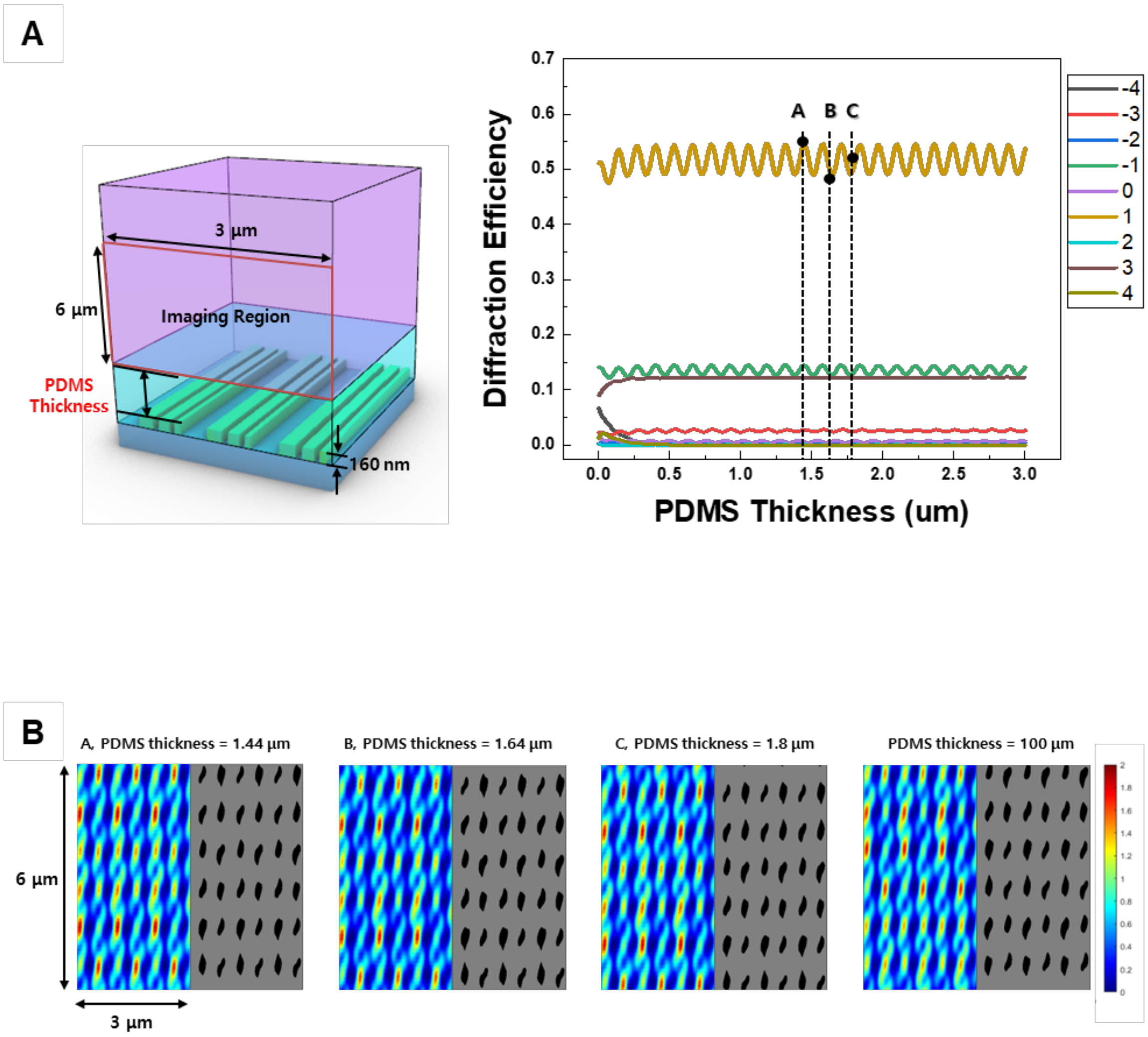

Fig. S11. Diffraction of an inversely designed phase mask at a wavelength of 355 nm with variations in the PDMS elastomer thickness. (**A**) Schematics of the diffraction (left) and diffraction efficiencies. (**B**) Images of the intensity distribution and cutoff structure (cutoff value: 0.15) at PDMS thicknesses of 1.44, 1.64, 1.8, and 100 µm.

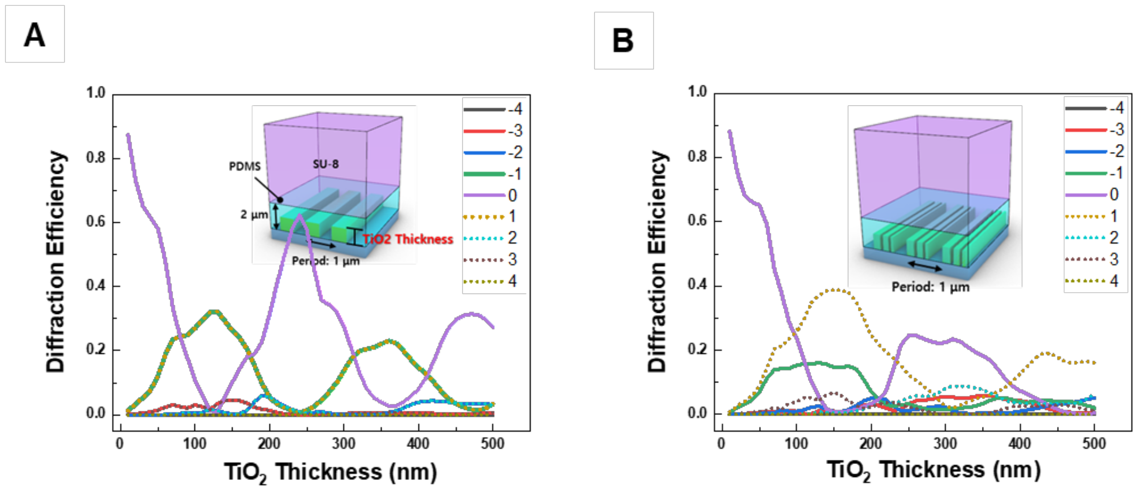

Fig. S12. Diffraction efficiencies of several orders for the TiO_2-inserted phase mask with a period of 1 µm at a 355 nm wavelength as a function of the TiO_2 thickness. (**A**) Simple line and space pattern (TiO_2 pattern width: 500 nm). (**B**) Inversely designed phase mask.

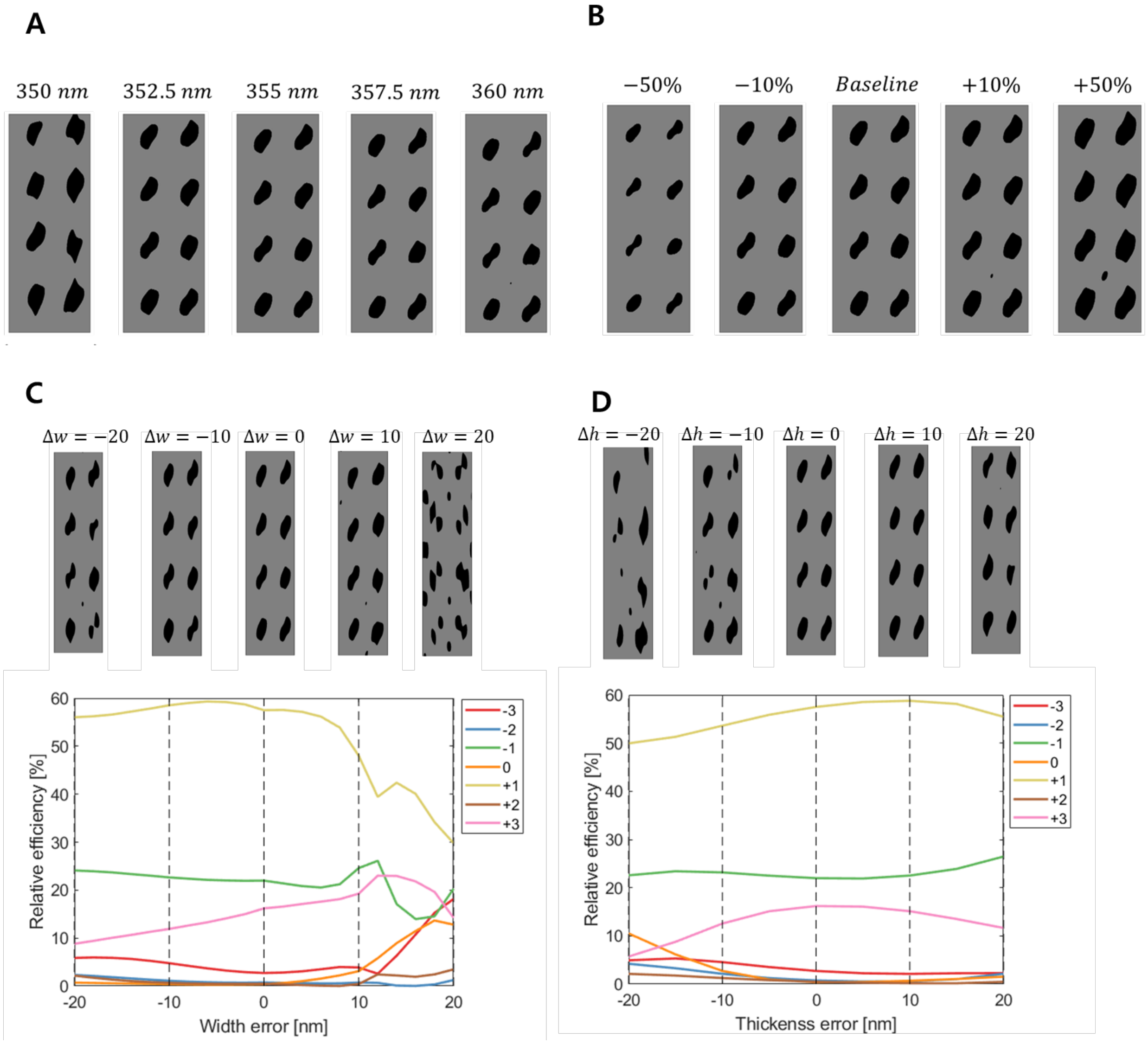

Fig. S13. Various sources of error for the inversely designed phase mask. (**A** to **B**) The patterns formed by wavelengths and intensities deviated from the predesigned value. (**C** to **D**) Relative efficiencies of each diffracted beam and corresponding patterns depend on width errors, and thickness errors, respectively.

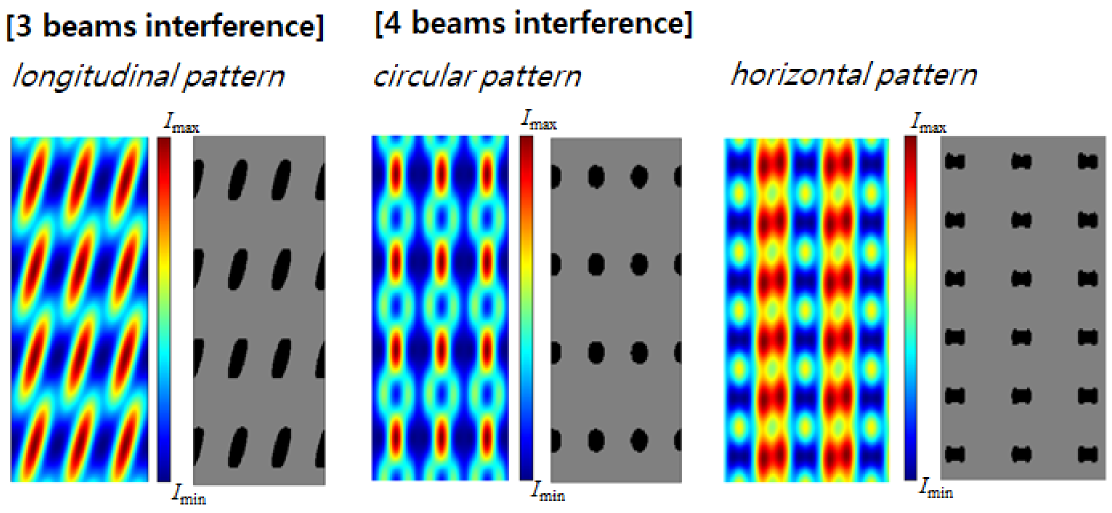

Fig. S14. Analytically calculated interference fields and corresponding patterns. (**A**) Longitudinal pattern created by three beams (-1st,1st,3rd). Phase mask period is set to 1 μm and photoresist refractive index is 1.66. (**B**) Circular pattern created by four beams (-3rd, -1st, 1st, 3rd). Phase mask period is set to 1 μm and photoresist refractive index is 1.66. (**C**) Horizontal pattern created by four beams (-3rd, -1st, 1st, 3rd). Phase mask period is set to 0.77 μm and photoresist refractive index is 1.41.

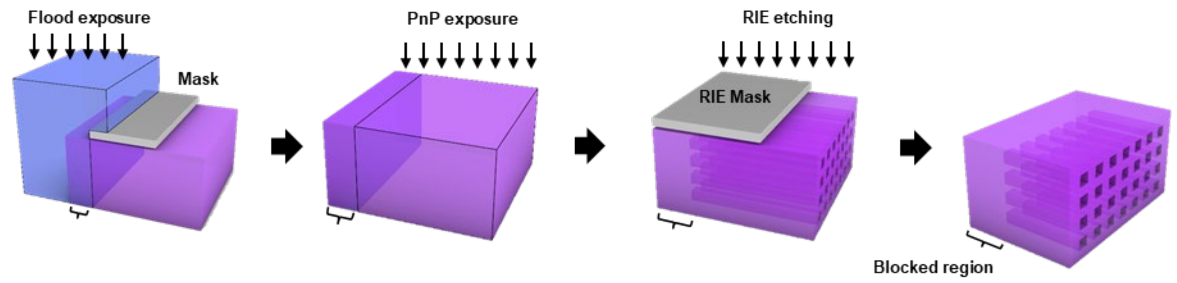

Fig. S15. Preparation of 2D nanochannel array with one end blocked *via* flood and PnP exposure and RIE etching.

Table S1. Design parameters and measured lengths in a period of SEM images in Fig. S10.

(unit: nm)	a	b	c	d	e	f
**Design**	455	196	50	185	62	52
**Si Master**	452	190	60	177	73	48
**PUA Mold**	445	191	69	170	84	41
**PMMA Pattern**	448	182	58	193	61	58
**TiO_2 Pattern**	443	198	65	190	58	46

Movie S1. Movie of inverse design of the PnP process. Each plot represents the relative efficiency, index profile, intensity of the corresponding pattern and field profile, as clockwise direction from top left.
